# Progress of metabolomics‐centric multi‐omics research in medicine

**DOI:** 10.1002/imo2.70096

**Published:** 2026-04-14

**Authors:** Ziyi Wang, Shiwen Deng, Xiaoxu Wang, Xiao Chang, Na Guo, Peng Chen, Hongjun Yang

**Affiliations:** ^1^ Experimental Research Center China Academy of Chinese Medical Sciences Beijing China; ^2^ AstraZeneca Pharmaceuticals LP Gaithersburg Maryland USA; ^3^ Department of Urology, The Third Medical Center Chinese People's Liberation Army (PLA) General Hospital Beijing China; ^4^ Analysis of complex effects of proprietary Chinese medicine Hunan Provincial Key Laboratory Yongzhou Hunan Province China

**Keywords:** artificial intelligence, medical research, metabolomics, multi‐omics integration, virtual cell

## Abstract

Metabolomics reveals molecular changes at the metabolic level during biological processes. Recent advancements, particularly through integration with other omics and artificial intelligence (AI), are advancing metabolic data analysis and multi‐omics interpretation. This review introduces a “metabolomics‐centric” paradigm as a conceptual framework for multi‐omics integration, positioning metabolic readouts as the functional anchor for interpreting genotype‐phenotype relationships. It also chronicles the evolution and medical applications of integration technologies. Our discussion first traces the field's evolution from bulk to single‐cell and spatial methodologies, next surveys integration strategies to uncover mechanistic insights, and finally examines how AI‐driven multimodal data integration and AI‐optimized metabolomics workflows tackle persistent biological and computational challenges. Looking forward, this review identifies the AI virtual cell as a pivotal future direction for metabolomics‐based multi‐omics integration, calling for increased attention to its development to simulate dynamic molecular phenotypic networks within a multi‐omics context.

## Highlights

- Proposes a “metabolomics‐centric” paradigm positioning metabolic readouts as the functional anchor for interpreting genotype‐phenotype relationships in multi‐omics integration.
- Surveys the technological evolution of metabolomics from bulk to single‐cell and spatial methodologies and their medical applications.
- Examines how artificial intelligence (AI)‐driven multimodal data integration and AI‐optimized workflows address persistent biological and computational challenges.
- Identifies the AI virtual cell as a pivotal future direction for metabolomics‐based multi‐omics integration toward predictive precision medicine.

## Graphical abstract

The graphical abstract illustrates a holistic roadmap for metabolomics‐centric multi‐omics integration in medical research. The upper panel depicts the technological transition from traditional bulk analysis to high‐resolution single‐cell and spatial methodologies, specifically addressing inherent challenges such as molecular complexity and dynamic variability. The right axis outlines the interactome layers, bridging genomic drivers with transcriptional and protein‐level metabolic networks. Crucially, the bottom section highlights the synergistic fusion of metabolomics and artificial intelligence (AI). By leveraging AI‐optimized workflows and multimodal data integration, this framework moves beyond descriptive analysis toward the development of an AI virtual cell. This integrated approach serves as a functional anchor to decode genotype‐phenotype relationships, ultimately advancing the implementation of predictive and precision medicine.

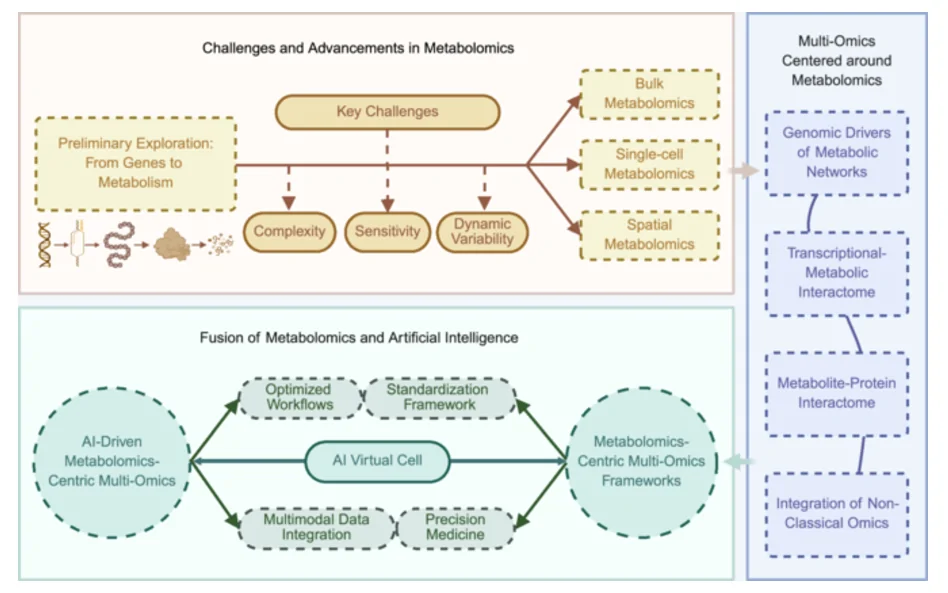

## 1 INTRODUCTION

Metabolomics, one of the youngest and most dynamic omics disciplines, is defined as the comprehensive analysis of low‐molecular‐weight metabolites within a biological system. These metabolites, as the functional endpoints of cellular processes, provide a direct snapshot of the physiological or pathological state, bridging genomic information with phenotypic manifestations [1, 2]. To capture this complex landscape, the field employs complementary strategies: untargeted, or discovery, metabolomics aims to broadly profile metabolites, while targeted metabolomics focuses on precise quantification of predefined pathways [3]. Specialized subfields, such as lipidomics, volatile metabolomics, urinary/serum metabolomics and pharmacometabolomics, address specific biological questions. Complementing these approaches, an emerging data‐driven strategy termed reverse metabolomics leverages specific mass spectrometry (MS)/MS spectra to mine public repositories, facilitating the discovery of novel phenotype‐metabolite associations in a hypothesis‐free manner [4]. Consequently, metabolomics finds broad utility in biomarker discovery, disease mechanism elucidation, and functional phenotyping, establishing itself as an indispensable tool for systems biology [5].

Compared to the upstream alterations in gene and protein expression, metabolomics provides a more direct depiction of the downstream functional readout of cellular physiological status. Initially closely aligned with clinical chemistry and biochemistry, the focus has shifted from basic metabolite detection toward a more comprehensive exploration of metabolic pathways and networks (Figure 1), which play important roles in cellular functions and disease mechanisms. The integration of metabolomics with other omics technologies offers a promising framework for systems biology. This multi‐omics approach enables researchers to correlate metabolic alterations with genetic, transcriptional, and proteomic changes, thereby facilitating dynamic biological processes, helping to elucidate disease mechanisms, and identifying potential biomarkers [6]. Nevertheless, integrating these multimodal data presents notable challenges, including handling multidimensional and voluminous datasets, variability across experimental platforms, and interpreting complex cross‐omics interactions.

**Figure 1:**
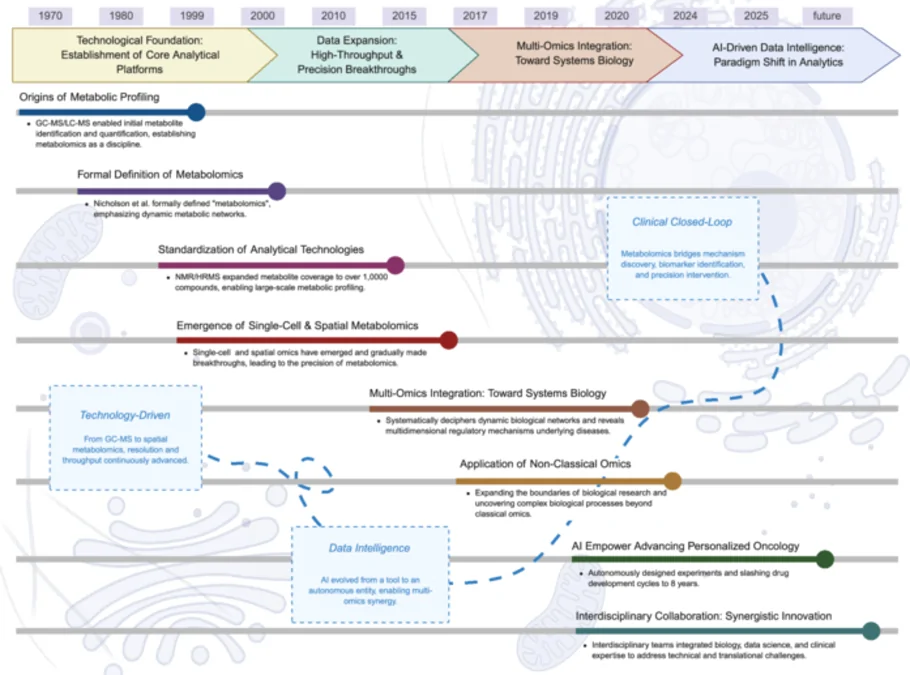
The evolution of metabolomics: paradigm shift toward multi‐omics integration. The development process of metabolomics presents a paradigm transition from technical foundation to multi‐dimensional integration. This technology evolution trajectory highlights the transformation of metabolomics from a single omics description to a dynamic system analysis and ultimately points to the personalized diagnosis and treatment goal of precision medicine. GC‐MS, gas chromatography‐MS; HRMS, high resolution mass spectrometry; LC‐MS, liquid chromatography‐mass spectrometry; NMR, nuclear magnetic resonance.

In recent years, artificial intelligence (AI) has provided promising new approaches for metabolomics to solve these challenges [7]. AI tools have the potential to enhance the scale and efficiency of metabolic data analysis and facilitate the integration and interpretation of multi‐omics data. When applied effectively, these approaches can improve the efficiency of data processing across various contexts [7, 8]. Moreover, AI‐driven integration of metabolic signatures with other omics layers is being actively explored for its potential to yield clinical benefits, such as aiding in early detection and treatment guidance [9]. This underscores the prospective value of metabolomics as a non‐invasive tool for precision medicine that links downstream products to phenotypic variation. Nevertheless, several challenges remain in achieving robust and scalable integration, particularly in reconciling data heterogeneity and ensuring biologically meaningful interpretation. The integration of multi‐omics data itself provides a critical foundation for the development of more sophisticated computational models, particularly AI virtual cell (AIVC) technology. By supplying comprehensive molecular profiles spanning genomics, transcriptomics, proteomics, and metabolomics, multi‐omics approaches enable the construction of spatially and temporally resolved models, thereby offering a dynamic and holistic representation of cellular metabolism [10].

This review examines the role of metabolomics in multi‐omics research, with a particular emphasis on its integration with AI. It traces the historical development and key technological milestones of metabolomics while critically examining the challenges and opportunities associated with its incorporation into multi‐omics workflows. Additionally, it highlights AI's contributions to enhancing the interpretation of metabolomics data and explores the application of metabolomics‐centric multi‐omics frameworks. In summary, it is crucial to place greater emphasis on the metabolomics‐centric integration of multi‐omics data and prioritize the development of AIVC technology.

## 2 THE EVOLVING LANDSCAPE OF METABOLOMICS METHODS

The vision for biology‐logic‐driven integration rests upon the foundational evolution of metabolomic technologies. We now trace this progression, from its origins, bridging genomics to phenotypes to the current paradigm where selecting the appropriate analytical strategies and resolution is a critical decision in experimental design.

### Bridging genotype to phenotype: The unique value and challenges of metabolomics

Metabolomics is a field that rose to prominence in the early 21st century as scientists sought to bridge molecular profiles, including genomics, transcriptomics, and proteomics, with functional cellular and organismal phenotypes (Table 1). This interdisciplinary convergence has established metabolomics as an important endpoint in the omics cascade, since metabolites most directly reflect cellular activity and physiological status. The field has undergone remarkable and sustained expansion throughout this period. Analyses of publication trends within omics have indicated that metabolomics has maintained a comparatively high growth trajectory, reflecting its increasing centrality to systems biology. This ongoing dynamism not only highlights growing interest but also supports the assertion, notably by computational biologists such as Theodore Alexandrov at the European Molecular Biology Laboratory, that metabolomics delivers the closest proxy to phenotypic expression [11]. Consequently, the rapid advancement of metabolomic technologies and methodologies has catalyzed renewed research focus on metabolic pathways and the essential roles of small molecules across diverse biological processes.

**Table 1:** Comparison of emphasis between metabolomics and other omics.

Omics type	Research focus	Key techniques	Primary applications	Advantages	Limitations
Metabolomics	Small molecules	GC‐MS, LC‐MS, NMR	Disease mechanism, biomarker discovery, and drug toxicity	Direct phenotypic reflection Cross‐species compatibility High sensitivity to dynamics	Complex metabolite annotation Limited coverage due to dynamic range
Genomics	DNA sequences and genetic variations	WGS, SNP array	Genetic disease diagnosis, driver gene identification	Reveals the genetic basis High data stability	Limited dynamic insights Incomplete functional annotation
Transcriptomics	mRNA expression profiles	RNA sequencing, microarray	Gene expression regulation, disease subtyping	Captures expression dynamics Identifies novel transcripts	mRNA‐protein expression discordance No post‐translational modification data
Proteomics	Protein expression and modifications	MS, antibody arrays	Protein function, drug target validation	Direct functional linkage Detects post‐translational modifications	Limited sensitivity for low‐abundance proteins High data complexity

Abbreviations: GC‐MS, gas chromatography‐MS; LC‐MS, liquid chromatography‐mass spectrometry; NMR, nuclear magnetic resonance; WGS, whole‐genome sequencing; SNP, single‐nucleotide polymorphism.

The canonical metabolomics workflow encompasses sampling, metabolite detection, and data analysis. Each stage is critical for generating high‐quality, biologically interpretable data. A major obstacle in establishing robust biomarkers stems from biases introduced during experimental design and execution [4]. These issues are further amplified when transitioning from data production to biological interpretation. Although direct quantitative profiling of metabolites in biofluids such as urine, saliva, serum, and tissues remains a central objective, the metabolome presents intrinsic complexity and a dynamic nature, characterized by vast chemical diversity and temporal fluctuation. These properties pose substantial challenges, effectively rendering the metabolome a “big, moving target” for investigators.

A great challenge in metabolite identification lies in distinguishing structural isomers among highly complex molecules. In contrast to the genome or the proteome, the metabolome consists of over a million compounds spanning numerous chemical classes. To extract meaningful information from such complex data, multivariate statistical methods such as Partial Least Squares‐Discriminant Analysis (PLS‐DA) and Orthogonal PLS‐DA are routinely employed. These techniques interpret metabolic shifts by maximizing inter‐group variance and help pinpoint significant metabolites based on variable importance and statistical threshold. Contemporary extensions of these models continue to address persistent issues such as high dimensionality, multicollinearity, and biological variability [12].

While these methodological advances have addressed several statistical and analytical challenges, the inherently dynamic nature of the metabolome introduces further complexity. Metabolite abundances fluctuate dramatically across spatial contexts and time scales, often changing within milliseconds, far more rapidly than transcripts or proteins. Although conventional multivariate tools detect patterns in bulk data, they have limited capacity to resolve single‐cell heterogeneity. This gap has spurred the development of single‐cell metabolomic technologies, which aim to resolve spatiotemporal metabolic dynamics in detail. Yet, achieving high resolution remains difficult due to extreme microenvironmental variability, which demands exceptional detection sensitivity. This requirement is further complicated by a fundamental constraint: unlike RNA, metabolites cannot be experimentally amplified.

### Integrated metabolic profiling: Biomarker discovery from analytical strategies to spatial resolution

The design of a metabolomics study is fundamentally guided by two strategic choices: the analytical approach for metabolite detection and the spatial resolution of the sample. The analytical approach is primarily divided into untargeted and targeted methodologies, which represent a trade‐off between coverage and precision. Untargeted metabolomics is commonly employed when the metabolites of interest are uncertain or unknown, enabling a hypothesis‐free, comprehensive analysis of the metabolic landscape. In contrast, targeted metabolomics is utilized when specific metabolic pathways are under investigation, prized for its high sensitivity, reproducibility, and capability for precise quantification [13]. This framework is essential for interpreting data across different spatial resolutions.

Driven by the inherent limitations of conventional bulk methods in resolving cellular heterogeneity, the field has progressively evolved in its spatial resolution. Bulk metabolomics, which analyzes population‐averaged samples from sources like blood, urine, or homogenized tissues, provides a crucial system‐level profile of metabolic states. The choice of analytical strategy here is critical: untargeted bulk profiling serves as the principal tool for initial, high‐throughput biomarker discovery due to its broad coverage, while targeted bulk methods are subsequently applied for rigorous validation and absolute quantification of candidate metabolites [14]. A common drawback, however, is the masking of cellular heterogeneity through population averaging, which can obscure critical metabolic diversity in complex systems like tumors [15]. To bridge the gap between coverage and precision, hybrid strategies such as widely targeted metabolomics have been developed, combining the broad scope of untargeted methods with the accuracy of targeted assays [16].

In contrast, the advent of single‐cell and spatial metabolomics has shifted the focus toward high‐resolution contextual analysis. Methodological improvements in single‐cell isolation and analytical platforms have substantially enhanced metabolic profiling at cellular resolution, even amid persistent challenges in sample preparation and technical limitations [17]. Advances in ionization technology, such as gas cluster ion beam secondary ion MS, which employs water cluster ion beams to reduce molecular fragmentation, have been instrumental in achieving near‐single‐cell spatial resolution [18]. Parallel innovations in sample preparation enable deeper metabolic profiling, exemplified by integrated platforms combining matrix‐assisted laser desorption/ionization (MALDI)‐MS with immunocytochemistry. Such approaches allow high‐throughput MALDI‐MS on unfixed cells to preserve native lipid and metabolite signals, followed by matrix removal and immunocytochemistry‐based cell typing [19]. This capability is especially valuable for uncovering metabolic heterogeneity within seemingly uniform cell populations, offering insights into cancer metabolism and other diseases driven by cellular diversity [20].

Building on these advances, the emergence of spatial metabolomics further extends resolution to the subcellular dimension. By integrating mass spectrometry imaging (MSI) with metabolomic profiling, spatial metabolomics enables the mapping of metabolite distributions within intact tissue architectures [21]. This field has progressed through techniques such as MALDI, desorption electrospray ionization (DESI), and notably air‐flow‐assisted DSEI‐MSI and MALDI‐MSI, which provide high spatial resolution, broad metabolite coverage, and large‐field imaging capabilities [22]. Similarly, SpaceM combines MALDI‐MS with fluorescence microscopy to achieve spatial metabolomics in over 10,000 cells [23]. High‐resolution methods like Spatial Single Nuclear Metabolomics, with pixel sizes around 1.5 µm, enable analysis of nuclear metabolomes and have been used to distinguish hepatocyte subpopulations in fibrotic liver tissues, linking subcellular metabolic phenotypes to disease mechanisms [24]. These technologies collectively facilitate in situ visualization and localization of metabolites, revealing spatial variations across tissue regions and cell types. For instance, Paul Kiessling et al. [25] present recent advancements in spatial multi‐omics technologies, emphasizing the integration of diverse omics data types to investigate molecular mechanisms in cardiovascular diseases, and underscoring the role of computational tools in translating spatial data into biological insights.

The translational impact of integrated approaches is particularly evident in oncology. MALDI‐MSI identified sphingolipid dysregulation in *HER2+* tumors that predicts response to trastuzumab [26], while ^13^C‐SpaceM revealed heterogeneity in fatty acid flux within the tumor microenvironment [27]. Such studies underscore how spatial resolution can uncover metabolic biomarkers with clinical utility. Integrative frameworks such as scSpaMet further combine non‐targeted spatial metabolomics with targeted multiplexed protein imaging on the same tissue section, enabling simultaneous quantification of metabolites and immunophenotypes at single‐cell resolution. This has revealed cell‐type‐specific metabolic states and local metabolite competition influenced by spatial factors such as vascular proximity in tumors [16]. The example highlights how the untargeted versus targeted paradigm extends into high‐resolution spatial and single‐cell studies, where an untargeted discovery phase can be followed by highly specific, targeted validation even within complex tissue architectures.

These technological developments pave the way for more precise biomarker discovery and also generate complex, high‐dimensional data that require sophisticated bioinformatic tools for interpretation. Standardized computational workflows, covering raw data preprocessing, normalization, batch correction, and dimensionality reduction, are therefore critical for cross‐platform reproducibility and biological inference. Many of these pipelines are available as open‐source software on GitHub, with containerized deployment options to ensure reproducibility and ease of adoption, contributing to a new era of standardized and collaborative metabolomics.

In conclusion, the choice among bulk, single‐cell, and spatial metabolomics should be guided by specific research objectives (Table 2). Critically, within the bulk profiling context, untargeted metabolomics is uniquely suited for initial, high‐throughput biomarker screening. Its scientific justification lies in its unbiased, hypothesis‐free nature, which provides a comprehensive view of the metabolic landscape and is essential for discovering novel and unexpected alterations [13], a decisive advantage over targeted approaches in the discovery phase. While cost‐effectiveness is a practical benefit, it is this capacity for global profiling that makes untargeted bulk analysis the foundation of biomarker discovery pipelines. Single‐cell approaches are essential for deciphering cellular heterogeneity and functional diversity, whereas spatial metabolomics is uniquely suited for mapping tissue‐scale metabolic gradients and microenvironmental crosstalk. A sequential integrative strategy often proves effective: employing untargeted bulk analysis to identify candidate pathways, followed by targeted assays for precise validation, and finally utilizing single‐cell or spatial resolution to localize biomarkers to specific cell populations and microanatomical contexts. The synergistic integration of these complementary approaches and analytical strategies will accelerate the discovery of spatially resolved, clinically relevant biomarkers.

**Table 2:** Comparative analysis of bulk, single‐cell, and spatial metabolomics technologies.

Characteristics	Bulk metabolomics	Single‐cell metabolomics	Spatial metabolomics
Throughput	High	Low	Medium
	Processes pooled samples or large cohorts efficiently	Limited by single‐cell isolation/detection sensitivity Requires high replication	Throughput depends on imaging speed/array density
Resolution	Tissue‐level	Single‐cell level	Spatial micron‐scale
	Averages metabolite signals across cell populations	Resolves intercellular heterogeneity	MALDI‐MSI: 20–50 μm DESI‐MSI: 50–200 μm SIMS: <1 μm
Coverage	Broad (1000+ metabolites)	Limited (50–200 metabolites)	Moderate (200–500 metabolites)
	Mature LC‐MS/GC‐MS/NMR platforms	Sensitivity constraints reduce detectable metabolites	Coverage varies by platform
Clinical Utility	Established	Emerging	Translational
	Biomarker discovery Diagnostic/prognostic assays	Rare cell subpopulations Precision therapy guidance	Tumor heterogeneity mapping Metabolic boundary analysis Drug distribution
Key Advantages	High coverage Low cost Standardized protocols	Unmasks cellular heterogeneity	Preserves spatial context Integrates with histopathology
Major Challenges	Loss of spatial/cellular information Insensitive to rare cells	Low sensitivity Analytical noise High cost	Resolution‐coverage trade‐off Complex data analysis

Abbreviations: DESI‐MSI, desorption electrospray ionization mass spectrometry imaging; MALDI‐MSI, matrix‐assisted laser desorption/ionization mass spectrometry imaging; SIMS, secondary ion MS.

## 3 MULTI‐OMICS INTEGRATION CENTERED ON METABOLOMICS FOR MEDICINE RESEARCH

Metabolomics serves as both a key readout and a functional regulator in diverse physiological processes. Because metabolite changes directly mirror an organism's physiological and disease states, they provide a critical bridge connecting genes, proteins, and phenotypes. As such, metabolomics has rapidly become integrated with other omics technologies, establishing itself as a critical element in systems biology and a driving force in modern disease research. The combined application of metabolomics with genomics, transcriptomics, proteomics, and other omics disciplines not only broadens our understanding of disease mechanisms but also provides comprehensive data support for precision medicine and treatment.

### Genomic drivers of metabolic networks: Deciphering phenotypic variations through genetic insights

Research into the integration of genomics and metabolomics has advanced notably in several interconnected areas: technical convergence across biological scales, functional validation through disease modeling, and advances in spatial resolution. This integrated approach builds upon the pan‐genomic framework, which redefined our understanding of species‐level genetic diversity [28, 29], and now operates at the critical interface between genotypic variation and phenotypic manifestation [30]. A key advantage of integrated omics is highlighted in recent methodological comparisons: whereas early studies analyzed genomic or metabolomic data separately [31], contemporary research increasingly leverages their synergistic potential. This shift mirrors the broader evolution from single‐reference genomics to pan‐genomic frameworks [17, 18], wherein comprehensive biological understanding necessitates multi‐dimensional data integration. The power of this technical synergy is exemplified in natural product discovery, where Caesar et al. [32] demonstrated that over 80% of compounds identified through integrated approaches are nonribosomal peptides or polyketides.

In oncology, this integration paradigm has enabled mechanistic dissection of metabolic reprogramming, a hallmark of malignancy [31]. Chen et al. [33] systematically mapped 94 effector genes to 109 metabolic alterations, while Kim et al. [34] revealed *KRAS/LKB1* co‐mutations induce *GFPT2*‐dependent hexosamine biosynthesis in non‐small‐cell lung cancer. These findings collectively validate the hypothesis that genomic‐metabolic integration can bridge genetic predisposition with functional pathophysiology [35].

Beyond cancer, integrated genomics‐metabolomics approaches have shown significant diagnostic potential in neurodegenerative and metabolic diseases. Schlosser et al. [36] identified 1299 metabolite‐genetic associations across body compartments, and Han et al. [37] established causal links between plasma metabolites and age‐related macular degeneration risk through metabolite genome‐wide association studies. Further expanding this approach, the role of metabolites in complex diseases like Alzheimer's disease (AD) has also been explored through genome‐wide association studies. For instance, Liu et al. [38] performed a large AD metabolome‐wide association study by developing blood metabolite genetic prediction models and providing new insights into the etiology of AD that involves blood metabolites and gut microbiome. Additionally, kidney disease studies by Montemayor et al. [39] and Wu et al. [40] further demonstrate the translational value of metabolite risk scores in clinical stratification.

The current frontier of integration extends to spatial resolution, where Sun et al. [41] and Ganesh et al. [42] have pioneered 3D multi‐omics mapping in gastric cancer and immune microenvironments, respectively. These spatially resolved methodologies not only validate previous genomic‐metabolic correlations but also uncover novel compartment‐specific interactions. Together, these advancements underscore that a comprehensive biological understanding requires both horizontal integration across omics layers and vertical resolution across spatial dimensions.

### Transcriptional‐Metabolic interactome: Decoding spatiotemporal dynamics across biological scales

The integration of transcriptomics and metabolomics has been propelled by three synergistic developments: technological convergence enabling multimodal measurement, cross‐disease mechanistic discovery, and functional network modeling. This paradigm is grounded in the principle that transcriptional regulation mediates metabolic adaptation [43], and recent methodological breakthroughs now allow simultaneous spatial resolution of both molecular layers [44].

Technical integration has advanced primarily through enhancements in spatial resolution. Vicari et al. [44] achieved single‐section integration of spatially resolved transcriptomics and MALDI‐MSI with under 5% signal interference, markedly improving upon the 15% cross‐modality noise in earlier protocols and addressing the critical need for preserved molecular context in neurodegenerative disease research. These technical advancements enable large‐scale systematic mapping, as demonstrated by Benedetti et al. [45], who constructed the Cancer Atlas of Metabolic Profiles across 11 malignancies, showing that 68% of metabolite variance covaried with immune‐related transcripts. This systematic mapping validates complex multi‐omics interaction networks and marks the paradigm's evolution from technical demonstration to a robust biological discovery engine.

Beyond oncology, this integrated approach yields unique pathomechanistic insights. Arif et al. [46] revealed two core gene‐metabolite subnetworks governing pulmonary function decline, demonstrating the predictive power of cross‐omics integration. In psychiatric applications, distinct computational strategies are required. Spathopoulou et al. [47] developed gradient‐boosted neural networks to integrate schizophrenia metabolomic signatures with single‐cell transcriptomes, overcoming unique challenges such as the blood‐brain barrier, which are absent in cancer studies. And as Benedetti et al. [45] conclusively showed, only 9.3% of gene‐metabolite correlations translate to druggable pathways, a gap that next‐generation multi‐omics must urgently address.

### Metabolite‐protein interactome: Systems‐level dissection of dynamic regulatory networks

The trajectory of proteometabolomic integration can be understood through distinct developmental phases. Early proof‐of‐concept studies focused on biomarker discovery [48], whereas contemporary research prioritizes mechanistic deconvolution through the lens of spatial resolution [49]. This progression generally mirrors a broader field‐wide shift from correlative analysis to causal inference, with methodological innovations bridging the gap between metabolite detection and functional proteomic validation [50].

The integration of proteomics and metabolomics is increasingly important in systems biology, supported by several interconnected strengths: (1) technical advancements enabling dynamic interaction mapping, (2) mechanistic elucidation of disease processes, and (3) translational applications spanning drug discovery to traditional medicine. This integration capitalizes on proteins as functional effectors and metabolites as dynamic mediators, thereby enabling a causal inference framework that surpasses single‐omics correlative analyses [50, 51].

Technical foundations were laid through innovative interaction mapping strategies. Piazza et al. [50] pioneered the LiP‐SMap workflow, which combines limited proteolysis with MS to quantitatively profile over 1500 metabolite‐protein interactions with an 85% validation rate, thereby revealing unprecedented enzyme promiscuity patterns. This methodology has been further refined through spatial resolution. Hu et al. [16, 49] demonstrated single‐cell spatially resolved metabolism, achieving 5 μm resolution in liver tissues, identifying 23 cell‐type‐specific interaction networks.

Cardiovascular research exemplifies the clinical translation of this paradigm. Previs et al. [52] used integrated proteometabolomic analysis to identify nucleotide deficiency and impaired fatty acid oxidation as hallmarks of hypertrophic cardiomyopathy. Building on this, Li et al. [53] decoded heart failure subtypes using deep myocardial proteometabolomic profiling, achieving 92% diagnostic accuracy through machine learning (ML) integration.

The approach's versatility extends to drug discovery and beyond. van Vugt et al. [51] mapped 35 cardiac risk metabolites to 38 druggable targets using plasma multi‐omics, with 6 candidates entering preclinical validation. Similarly, in infectious diseases, Bi et al. [54] established urinary proteometabolomic signatures predicting COVID‐19 severity, validating the paradigm's diagnostic versatility.

Notably, the integration of metabolomics and proteomics has also been effectively applied in the field of traditional medicine for resource identification and quality assessment. For instance, Sun et al. [55] employed non‐targeted metabolomics and label‐free proteomics to explore the metabolic and proteomic differences between two types of *Scutellaria baicalensis*. Their results documented significant changes in metabolite accumulation and protein expression, particularly in pathways related to flavonoid biosynthesis and amino acid metabolism. This work not only provides insights into the distinctions between the two types but also offers guidance for selecting the optimal harvest period, demonstrating the utility of multi‐omics integration in traditional pharmacognosy.

### Integration of non‐classical omics with metabolomics: Expanding perspectives and cross‐disciplinary applications

Integrated multi‐omics approaches, particularly coupling metabolomics with emerging non‐classical omics fields, provide unprecedented mechanistic insights into complex biological systems and disease pathogenesis. Moving beyond classical genomics, proteomics, and transcriptomics, these non‐classical omics broaden the interpretative scope and functional context of metabolomics research, enabling cross‐disciplinary applications and supplying novel impetus for understanding physiological phenomena, disease mechanisms, and clinical translation through multi‐dimensional data.

#### Glycomics (Glycoproteomics)

Glycomics identifies glycome structure, function, and governing factors, demanding robust, sensitive, high‐throughput analytical technologies [56]. As aberrant glycosylation marks cancer, glycoproteomics is vital for biomarker discovery. Sun et al. [57] employed tandem mass tag labeling with a novel chemical labeling‐assisted dissociation method for multiplexed, site‐specific N‐glycosylation profiling of human serum IgG. Analyzing 90 patient sera, they identified distinct IgG glycoforms as potential liver disease staging biomarkers, validated in an independent cohort. In some metabolic diseases, multi‐omics analysis traces information flow from genomics to epigenomics, transcriptomics, proteomics, metabolomics, and glycomics, deepening understanding of pathophysiology [58]. MS advances facilitate this integration, enabling simultaneous detection of thousands of metabolic features and complex glycoproteomic analysis within a single platform [5].

#### Microbiomics

Microbiomics elucidates gut dysbiosis‐disease relationships by analyzing microbiota composition and function [59]. Recognizing metabolomics as crucial to microbiome research, the field shifted from taxonomic genomics toward integrating metabolomics and metagenomics [60, 61].

Integrated analyses systematically investigate gut microbiota and their metabolic products, clarifying microbial impacts on host physiology. For example, microbiota‐derived secondary bile acids may regulate host lipid metabolism via the farnesoid X receptor activation [62]. A meta‐analysis of 10 human gut studies used ML to identify 97 “robustly well‐predicted” metabolites, including those involved in bile acid and polyamine metabolism [63]. Modern computational methods integrate metagenomic and metabolomic datasets, identifying microbial taxa and genes driving metabolite variations [64, 65]. Key tools include: MIMOSA2 [66], linking metabolites to microbial taxa and mechanisms [67]; MMVEC [68], predicting metabolite abundance from microbial sequences using neural networks [65]; and the DMoVGPE model [69, 70] addressing integration challenges. The field advances toward single‐cell resolution using microfluidics [69, 71] and spatial analysis via techniques like spatial meta‐transcriptomics, mapping host‐microbe interactions in tissue regions [72]. Notably, metabolite predictability is often study‐specific, reflecting microbiome‐metabolite interaction heterogeneity across populations [73].

#### Exposomics

Exposomics studies the exposome, including cumulative lifetime environmental exposures and biological responses [74, 75]. Proposed by Wild in 2005, it aims to bridge environmental health research gaps using comprehensive, life‐course perspectives and multi‐omics integration [76]. Novel LC‐high resolution MS‐based exposomics approaches complement metabolomics, enabling semi‐quantitative perfluoroalkyl and polyfluoroalkyl substances precursor detection and elucidation of their interactions with endogenous metabolism [77]. The Chemical Correlation Database catalogs inter‐chemical correlations in metabolomics/exposomics data, aiding interpretation of xenobiotics and endogenous metabolites across 35 human studies [78]. Epigenomics bridges environmental exposures and metabolic regulation, as modifications are influenced by external factors and modulate metabolic enzyme expression [58]. Integrated genomic, epigenomic, and metabolomic data in nonalcoholic fatty liver revealed that environmental toxins drive lipid accumulation and inflammation by epigenetically silencing metabolism genes [58]. Similarly, multi‐omics in COVID‐19 showed the virus exacerbates damage by disrupting host epigenetic regulation and metabolic reprogramming [79].

#### Radiomics

Radiomics extracts high‐throughput quantitative features from medical images to enhance diagnostic, prognostic, and predictive accuracy [80], focusing on complex, reproducible patterns beyond human recognition [81]. Recently, international collaboration standardized key convolutional filters, producing reference images and feature values to enhance consistency across radiomics platforms and support clinical decision systems and deep learning (DL) [82].

### Metabolomics‐centric multi‐omics frameworks: Strategies in multi‐modal data integration and precision disease profiling

#### Multi‐dimensional integration under the standardization framework: Decoding biological complexity across scales

The inherent complexity of biological systems necessitates integrative multi‐omics approaches to decode molecular interactions. Metabolomics serves as a crucial component within multi‐omics integration (Figure 2) by delivering a proximate readout of cellular phenotypes and physiological states, thereby functionally contextualizing other omics layers to create comprehensive molecular profiles. However, multi‐omics integration extends beyond mere data aggregation and critically depends on harmonizing multi‐layered omics data from the same tissue. The increasing complexity and dimensionality of datasets necessitate robust computational methods to handle heterogeneous data types and large sample sizes [83, 84]. Furthermore, there is often a gap between the pace of data generation and the ability to process and analyze these datasets effectively [85]. Standardization of analytical pipelines, data preprocessing, and result interpretation remains an active area of development [86]. The maturity of different omics technologies is uneven, and the field requires multidisciplinary expertise spanning biology, statistics, and computer science to ensure successful integration and meaningful biological interpretation.

**Figure 2:**
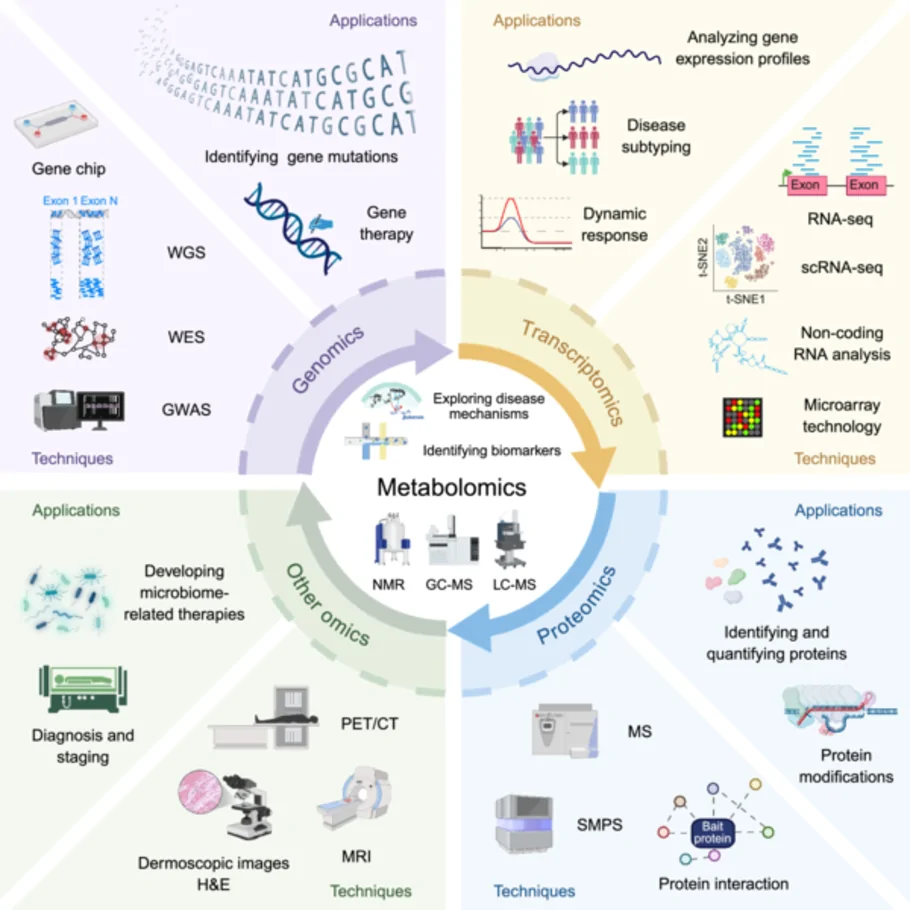
Multi‐omics integration in disease research. A Metabolomics‐Centric Framework. These integrated layers decode disease mechanisms, identify biomarkers, and inform therapeutic strategies, advancing precision medicine through dynamic, system‐level insights. GWAS. genome‐wide association study; H&E, hematoxylin and eosin staining; MRI, magnetic resonance imaging; PET/CT, positron emission tomography/computed tomography; Seq, sequencing; SMPS, single‐molecule protein sequencing; WGS, whole genome sequencing; WES, whole exome sequencing.

A primary hurdle to standardization is the inherent variability in consortium‐driven datasets, which often suffer from platform‐, batch‐, and lab‐specific variations that amplify analytical complexities and obscure true biological signals [87]. To overcome these issues, the field is advancing on two complementary fronts: establishing robust reference benchmarks and developing advanced computational harmonization methods. For the former, initiatives like the Quartet project provide matched multi‐omics reference materials from family cell lines, establishing critical benchmarks for data quality control and integration [87]. For the latter, methodological innovations prioritize computational harmonization. For instance, Pan et al. [88] proposed constructing multi‐omics heterogeneous networks to integrate multimodal data, thereby enhancing feature extraction and predictive accuracy in biomedical research.

To address these challenges, a variety of integration strategies have been developed, generally falling into three categories: network‐based, statistics‐based, and deep learning‐based methods [84]. Each approach employs different core algorithms and is suited to specific types of data and research questions (Table 3). Currently, a substantial array of tools is available, employing diverse methodologies to combine and analyze different types of omics data. Integration approaches have been reviewed from various perspectives [108], and thorough comparisons of major methodological families exist [109]. Prominent examples of these strategies include multi‐Omics Factor Analysis (MOFA), a widely applicable unsupervised method designed to discover the principal latent sources of variation across datasets [110], and Data Integration Analysis for Biomarker discovery using Latent variable approaches for Omics studies (DIABLO), a supervised integration framework within the mixOmics package [111]. Other tools, such as iClusterPlus and iClusterBayes, offer comprehensive analysis capabilities, albeit with greater computational demands [112]. Additionally, user‐friendly software platforms and graphical interfaces have been created to facilitate access for researchers without extensive computational backgrounds, although many advanced tools still require programming expertise and substantial computational resources [84, 86]. Ultimately, the selection of the most appropriate method or tool is contingent upon the research objective, the nature and quality of the data, and the available computational infrastructure.

**Table 3:** Methodological classification and translational evaluation of multi‐omics data integration tools.

Classification	Tool name	Core algorithm/approach	Strengths	Limitations	Exemplary medical/translational applications	Reference
Network‐Based Integration	SNF, NEMO, CIMLR, MultiNMF, PFA, OmNI	Constructs and fuses sample similarity networks, performs joint matrix factorization, or employs network analysis algorithms across omics views.	Robust to noise and effective at balancing shared and specific information across data types. Capable of identifying clinically relevant molecular subtypes with high accuracy. Some variants like PFA integrate prior biological knowledge, and newer tools like OmNI offer interactive network visualization and pathway enrichment to enhance interpretability.	Performance can be sensitive to parameters. Scalability may be challenged by very large cohort sizes. Biological interpretation of fused networks or factors often requires substantial downstream validation.	Cancer subtyping and prognosis prediction (e.g., via SNF, CIMLR). Patient stratification based on dysregulated pathway modules (e.g., PFA). Integrative biomarker discovery across omics layers (e.g., MultiNMF).	[84, 89, 90, 91, 92, 93]
Statistics & Matrix Factorization	mixOmics, MOFA, IClusterBayes, LRAcluster, moCluster, Holomics	Probabilistic modeling, PLS, or other latent variable methods to find shared subspaces.	Provides a unified statistical framework for diverse data types. Enables supervised or unsupervised analysis with feature selection for biomarker identification. Offers strong visualization and validation utilities.	Often assumes linear relationships; non‐linear extensions are less mature. Can be computationally intensive and require careful data pre‐processing. May involve a steep learning curve due to statistical and programming requirements.	Molecular subtyping of complex diseases (e.g., iClusterBayes). Biomarker discovery and validation for precision diagnosis (e.g., DIABLO in cancer).	[84, 94, 95]
DL‐Based	Subtype‐GAN, MOGONET, MORE, GLUE, Flexynesis, OmicsFootPrint	Advanced neural architectures for non‐linear integration, representation learning, and supervised prediction.	Superior capability in modeling complex, non‐linear relationships, with some tools integrating prior knowledge or converting data to images. Demonstrate state‐of‐the‐art performance in biomedical classification, survival, and drug response prediction. Potentially identifies novel disease‐relevant biomarkers, with improved interpretability via SHAP and modular designs for various tasks.	High demand for computational resources and advanced technical expertise. Proned to overfitting in small‐sample scenarios, affecting robustness. Limited model interpretability hinders biological insight extraction, though some tools incorporate explainability methods.	Precision classification, survival prediction, and drug response Novel subtype identification and integration with prior knowledge.	[96, 97, 98, 99, 100, 101]
User‐Friendly Platform	BiomiX, OmicsNet, MiBiOmics, Galaxy, Mergeomics, OmnibusX	Web‐based or desktop graphical interfaces that wrap underlying statistical or network algorithms into workflow‐driven, point‐and‐click environments, supporting diverse modalities.	Dramatically lowers the barrier to entry, enabling analysis without programming expertise; some platforms prioritize data privacy through local processing. Promotes reproducibility through shareable workflows and emphasizes interactive visualization for hypothesis generation. Suitable for exploratory analysis and specific domains, with support for cutting‐edge single‐cell multi‐omics.	Analytical depth and flexibility are often limited compared to code‐based packages. May have restrictions on data types, scale, or layers. Performance can be constrained by server resources or pre‐built module availability; desktop tools rely on local resources, which may limit large‐scale analysis.	Exploratory integration and visualization in translational research cohorts, including privacy‐preserving clinical single‐cell analysis. Pathway‐centric network analysis and enrichment. Reproducible workflow execution for standardized analyses.	[102, 103, 104, 105, 106, 107]

Abbreviations: CIMLR, cancer integration via multikernel learning; GAN, generative adversarial networks; LRAcluster, low‐rank approximation‐based multi‐omics data clustering; MOGONET, multi‐omics graph convolutional networks; MORE, multi‐omics representation learning; MultiNMF, multi‐view clustering via joint nonnegative matrix factorization; NEMO, neighborhood‐based multi‐omics clustering; OmNI, omics notebook interactive; PFA, pattern fusion analysis; SNF, similarity network fusion.

Meanwhile, several public repositories support multi‐omics research and applications. The Gene Expression Omnibus (GEO) is a comprehensive public repository housing functional genomics datasets submitted by the global research community, maintained by the National Center for Biotechnology Information. Multi‐omics data are increasingly integrated within GEO, enhancing their predictive power and facilitating the identification of novel biomarkers, key regulatory mechanisms, and potential therapeutic targets [113]. FaceBase provides access to diverse datasets relevant to human development and phenotypes, encompassing three primary data types: chemical data, clinical data, and molecular biology/biochemistry data [114].

Despite these resources, a significant technical gap persists: analytical tools and methods often remain isolated across omics layers, and achieving truly integrated, single‐cell resolution analysis remains challenging [115, 116]. To bridge this gap, technological innovations are emerging. For instance, methods like scMeT‐seq employ sub‐picoliter upgrade sampling to separately capture metabolites and RNA from the same single cell, enabling synchronous multi‐omics analysis at an unprecedented scale [117]. In a complementary manner, Ye et al. [118] systematized methods to identify molecular Quantitative Trait Loci across transcriptomic, epigenetic, proteomic, and metabolomic layers, enabling causal inference in metabolic regulation. These advances converge within frontier frameworks aimed at mapping regulatory networks with high resolution.

Although network‐based approaches represent a predominant strategy in most current multi‐omics studies [115], favored for their flexibility and direct integration capabilities, it is important to recognize that analytical tools and methods often remain isolated across omics layers. Significant technological innovation is still required to achieve single‐cell level multi‐omics analysis or to establish a unified framework ensuring reproducibility and biological relevance. Given the current proliferation of computational tools, we propose utilizing a “Metabolomics Integration Efficacy Assessment Matrix.” This matrix systematically evaluates existing technologies across four dimensions: resolution, throughput, dynamic range, and integration compatibility, assigning quantitative scores. This framework assists researchers in selecting the optimally suited analytical approach based on their specific requirements.

#### Metabolomics‐driven multi‐omics integration: Bridging molecular mechanisms to precision therapeutics

Metabolomics within multi‐omics provides a functional and dynamic lens through which to view the complexity of physiological responses to external events and conditions. Integrating these diverse omics layers enables researchers to collect and analyze data at scale from multiple clinical and molecular modalities. By elucidating the molecular mechanisms underpinning disease progression and drug efficacy, this integration fosters novel target identification and drug repurposing opportunities [119].

Metabolomics‐driven multi‐omics integration is increasingly recognized as a driving force in precision medicine, enabling more refined disease subtyping, especially within cancer research [112]. Kreutzer et al. [43] sequentially coupled MALDI‐MSI with whole exome sequencing and RNA‐seq on formalin‐fixed paraffin‐embedded sections of head and neck squamous cell carcinoma, enabling simultaneous exome, transcriptome, and spatially resolved metabolome analysis. This spatial multi‐omics approach has been further refined to address tissue heterogeneity. Li et al. [120] combined single‐cell ATAC/RNA sequencing with spatial metabolomics in kidney tissue, analyzing over 408,000 profiles, which unveiled previously unrecognized cellular diversity.

Multi‐omics approaches, by integrating orthogonal data layers, are increasingly contributing to the transformation of biomarker discovery and have emerged as a prominent strategy for deconstructing mechanisms across multiple diseases. Manav et al. [121] finely mapped complex loci to discover alcohol use disorder susceptibility genes at single‐SNP resolution, uncovering shared genetic mechanisms with neurodegenerative disorders. In breast cancer, whole metabolome‐wide association studies identified 41 candidate biomarkers through a prospective nested case‐control study [122]. For cardiometabolic disease risk assessment, the fusion of genomics, proteomics, and metabolomics has identified composite biomarkers that enhance predictive accuracy and mechanistic understanding. Leveraging transcriptomic, proteomic, and metabolomic profiling, Mikaeloff et al. [123] identified plasma biomarkers and metabolic disturbances associated with persistent inflammation, monocyte dysfunction, and neuropsychiatric risk, thereby clarifying immunometabolic complications in successfully treated people with human immunodeficiency virus (HIV) and providing a roadmap for precise interventions in HIV care. The pathway from integrated multi‐omics discovery to clinical application is contingent upon overcoming interconnected challenges in data processing, biological interpretation, and real‐world validation (Table 4).

**Table 4:** Representative studies demonstrating metabolomics‐centric multi‐omics integration for translational discoveries in precision medicine.

Research area	Integrated omics strategy (metabolomics +)	Key findings and integrative insights	Clinical/translational implications	Breakthroughs and remaining challenges	Examples
Pan‐tumor/Common solid tumors	Genomics, Transcriptomics, Proteomics	Metabolomics serves as a functional readout of dysregulated pathways driven by genomic alterations, revealing disease‐specific metabolic signatures, oncometabolites, and nutrient dependencies. Integration identifies metabolic vulnerabilities linked to oncogenic drivers and tumor microenvironment.	Informs precision oncology by enabling biomarker discovery, guiding the development of targeted metabolic therapies, and improving metabolic imaging interpretation.	Breakthroughs: Successfully demonstrates that multi‐omics integration can uncover actionable biological insights beyond single‐omics analyses, driving the discovery of novel therapeutic targets and diagnostic strategies. Challenges: Technical hurdles include data harmonization across heterogeneous platforms, incomplete metabolome coverage, difficulties in establishing causal relationships, and translating integrated findings into standardized, clinically validated tools.	[124]
AD	Genomics, Lipidomics, Proteomics, Microbiomics	Metabolomic and lipidomic dysregulation is integrated with genetic risk, gut microbiome shifts, and proteomic profiles, revealing systemic metabolic failure, neuroinflammation, and distinct molecular subtypes.	Enables blood‐based biomarker panels for early detection and progression prediction. Guides patient stratification for targeted therapies and supports drug repurposing.	Breakthroughs: AI‐driven integration of high‐dimensional datasets; identification of minimally invasive blood biomarkers; mechanistic linkage of gut‐brain axis to pathology; definition of biologically distinct patient subtypes. Challenges: Heterogeneity in data; establishing causal relationships from observational omics data; clinical validation of multi‐omics signatures in diverse, longitudinal cohorts; high computational cost and need for standardized, interoperable analysis pipelines.	[125, 126]
T1D	AI/ML‐powered integration of multi‐omics data (genomics, transcriptomics, proteomics, immunopeptidomics)	AI/ML models enable the discovery of novel drug targets, prediction of synergistic drug combinations, and identification of patient subgroups based on genetic and immune signatures. These approaches facilitate drug repurposing, optimize antigen‐specific immunotherapy design, and predict individual pharmacokinetic/pharmacodynamic profiles.	Accelerates precision medicine by enabling in silico prediction of optimal therapies and dosing, stratifying patients for clinical trials, and identifying biomarkers for early intervention. Aims to shorten trial timelines and improve therapeutic efficacy.	Breakthroughs: AI/ML processes high‐dimensional data to reveal non‐linear relationships, enabling predictive modeling of disease progression and treatment response at an unprecedented scale. Challenges: Requires high‐quality, diverse, and FAIR data; models often lack interpretability; risks of algorithmic bias; and necessitates rigorous clinical validation for real‐world implementation.	[127, 128]
T2D (Chinese, Malay, Indian)	Genomics, Transcriptomics, Epigenomics	Large‐scale multi‐ethnic Asian cohort reveals ethnicity‐specific genomic regulation and metabolic profiles linked to diabetes risk, highlighting population diversity in disease etiology.	Provides a foundational resource for ethnicity‐aware precision medicine in Asian populations. Enables identification of population‐specific biomarkers and therapeutic targets.	Breakthroughs: Largest multi‐ethnic Asian cohort with deep multi‐omics phenotyping; reveals population‐specific regulatory mechanisms. Challenges: Longitudinal validation needed; translation of population‐level insights to individual clinical tools requires further development.	[129]
T2D (Chinese)	Genomics	Integrated metabolome‐wide GWAS and Mendelian randomization identify causal metabolites linking T2D to cardiovascular complications in the Chinese population, with cross‐ancestry validation.	Identifies potential therapeutic targets and cross‐ancestry biomarkers for personalized prevention; emphasizes urea cycle pathway in cardiometabolic disease progression.	Breakthroughs: First high‐coverage metabolome‐wide GWAS in Chinese population with causal inference; demonstrates cross‐ancestry Mendelian randomization for biomarker validation. Challenges: Requires validation in larger cohorts; limited functional validation; translation to clinical practice needs standardized assays.	[130]
Epilepsy	Genomics, Transcriptomics, Proteomics, Metagenomics	Metabolomics serves as a downstream phenotypic readout, revealing functional consequences of genomic variants and network perturbations. Integrative analyses identify dysregulated pathways, specific metabolite alterations, and gut microbiome‐metabolome interactions linked to epileptogenesis and drug resistance.	Aids in discovering non‐invasive biomarkers for early diagnosis, patient stratification, and prognosis. Identifies novel therapeutic targets beyond ion channels. Supports personalized management strategies, such as predicting response to ketogenic diet based on multi‐omics signatures.	Breakthroughs: Provides a holistic, functional view of disease mechanisms across molecular layers; successfully identifies cross‐omics signatures associated with specific epilepsy subtypes or treatment outcomes; enables the discovery of modifiable factors for intervention. Challenges: Data integration complexity; lack of standardization; limited sample sizes and translational gap; high costs and technical expertise.	[131, 132]
Kidney diseases	Lipidomics/Glycomics, Transcriptomics, Proteomics, Epigenomics	Integrated multi‐omics identified key disease‐driving metabolites mapped to specific cell subtypes and injury regions, revealing cell‐type‐specific metabolic reprogramming, altered lipid/glycan landscapes, and linked dysregulated pathways underlying fibrosis and tumor biology.	Discovery of novel non‐invasive metabolic biomarkers for disease prediction/prognosis; enabled AI‐based diagnostic classification of disease subtypes; identified novel potential therapeutic targets within dysregulated metabolic networks.	Breakthroughs: High‐resolution spatial mapping preserves tissue context; computational platforms enable robust cross‐omics integration. Challenges: Comprehensive metabolite annotation remains limited; requires standardization of quantitative methods and data integration pipelines; translating discoveries into routine clinical practice faces technical and validation hurdles.	[133]

Abbreviations: GWAS, Genome‐Wide Association Studies; T1D, Type 1 diabetes; T2D, Type 2 diabetes.

In current clinical applications, metabolomics is particularly valuable for the dynamic monitoring of therapeutic interventions. Its ability to track metabolic reprogramming in real‐time provides critical insights into drug efficacy and resistance. For example, in oncology, adaptive changes in glycolysis and amino acid metabolism during treatment reveal tumor‐specific vulnerabilities, enabling clinicians to tailor therapies based on evolving metabolic profiles [134]. Complementing this, Wang et al. [135] developed a high‐resolution nuclear magnetic resonance relaxation method for continuous biofluid metabolomics monitoring, facilitating real‐time assessment of treatment progression and metabolic rebound.

Metabolomics‐driven approaches are expanding therapeutic frontiers beyond conventional drug discovery paradigms. A notable application lies in traditional Chinese Medicine (TCM), where metabolomics synergizes with TCM‐omics to evaluate herbal efficacy, identify bioactive compounds, and map metabolic pathways. Ren et al. [136] comprehensively reviewed these applications, emphasizing metabolomics' role in bridging empirical herbal knowledge with modern mechanistic validation. This integration not only revitalizes ancient pharmacopeias but also accelerates the discovery of novel targets and repurposing opportunities across diverse therapeutic areas. Furthermore, the confluence of metabolomics and microbiomics holds significant promise. For instance, based on findings that microbial and metabolic disturbances in myalgic encephalomyelitis/chronic fatigue syndrome exhibit stage‐specific patterns, Xiong et al. [137] linked these findings to mechanistic pathways involving butyrate and bile acid metabolism.

As multi‐omics studies continue to expand, metabolomics‐centric integration will remain key to deciphering the complex mechanisms of human diseases, unearthing biomarkers, and ultimately enabling clinical implementation to advance personalized medicine. However, clinical translation remains challenging, requiring rigorous validation and standardization to bridge the gap between research and routine practice [138]. While pushing the biomarker frontier, it also demands robust frameworks for clinical deployment.

## 4 AI‐DRIVEN METABOLOMICS‐CENTRIC MULTI‐OMICS INTEGRATION

The Royal Swedish Academy of Sciences has awarded the Nobel Prize in Physics 2024 to John J. Hopfield and Geoffrey Hinton for foundational discoveries and inventions that enable ML with artificial neural networks [139]. AI reshapes metabolomics through a vertically integrated framework spanning detection optimization, advanced data analytics, and dynamic visualization. These technological advancements mature into clinical translation via multi‐omics convergence, enabling precision diagnosis through biomarker discovery and molecular subtyping while empowering therapeutic precision via real‐time metabolic monitoring to guide targeted interventions (Figure 3).

**Figure 3:**
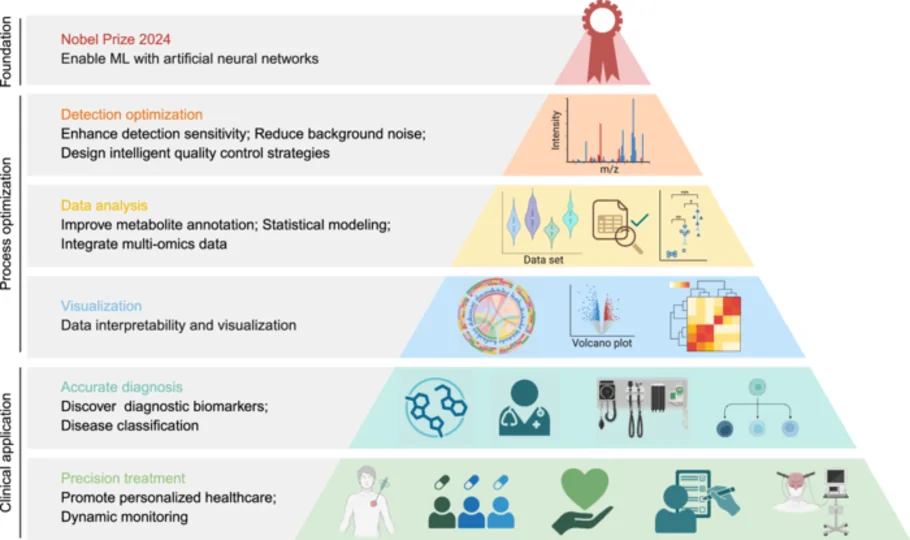
AI‐driven metabolomics: from neural network foundations to precision medicine implementation. Top: The 2024 Nobel Prize in Physics. Middle: Process optimization layers enabling metabolic breakthroughs (detection optimization, AI‐enhanced data analysis, and dynamic visualization). Base: Clinically validated applications advancing precision diagnosis and therapeutic tailoring. ML: machine learning.

### The imperative for AI‐driven metabolomics: Addressing necessities and overcoming challenges

The integration of AI with metabolomics has become increasingly vital for advancing multi‐omics research, driven by both the intrinsic complexities of metabolomic data and the growing demand for systems‐level biological insight.

A primary motivation for AI integration lies in handling the scale and complexity of metabolomic data. Metabolomics generates vast and complex datasets, with strong feature correlations and substantial noise [140]. AI techniques leverage powerful pattern recognition and data processing to help reveal underlying structures and relationships that facilitate biomarker discovery, disease classification, and metabolic network reconstruction [141]. Furthermore, AI enhances the integration of metabolomics with other omics layers through multimodal modeling, improving predictive accuracy and mechanistic interpretation [142], proving particularly valuable in precision medicine for applications such as cancer subtyping and treatment outcome prediction.

In clinical settings, metabolomics encounters additional difficulties, including limited analytical sensitivity, challenges in data standardization, and complex metabolite annotation [143]. AI contributes by helping automate data preprocessing, refining metabolite identification via knowledge graph‐assisted annotation, and developing standardized computational workflows [144]. It also demonstrates considerable promise in constructing predictive models for early disease diagnosis [145], monitoring treatment responses, and identifying novel drug targets [146]. An important benefit lies in its ability to distill high‐dimensional data into clinically actionable insights, supporting data‐driven decisions while reducing reliance on costly instrumentation [147].

Integrative metabolomics confronts ongoing challenges such as technical variability, biological heterogeneity, and mechanistic interpretation of cross‐omics interactions. These issues underscore the need for tailored computational frameworks and highlight the critical need for advanced computational approaches [115]. AI offers a promising pathway to address these limitations, enabling more robust integration and interpretation of metabolomics within multi‐omics research. The historical evolution of AI, from early machine intelligence concepts to modern deep learning and large language models (Figure 4A), has paved the way for its expanding applications across omics disciplines, including disease research and the progression toward autonomous systems (Figure 4B).

**Figure 4:**
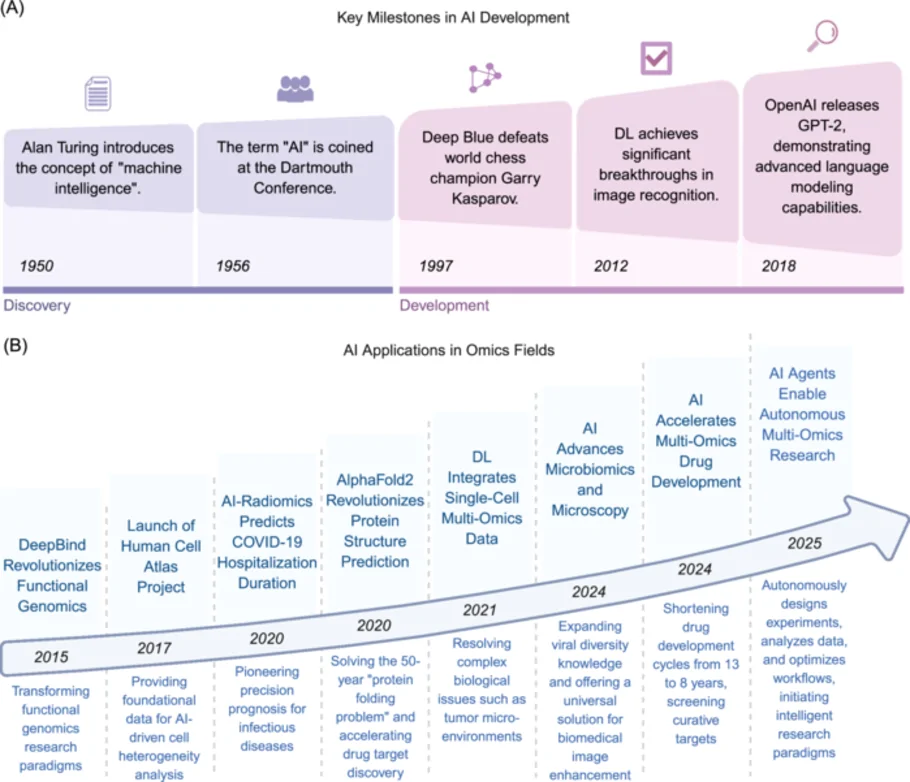
Key milestones in AI development and its application areas in omics fields. (A) Timeline of several major breakthroughs in AI, from early concepts of machine intelligence to the rise of DL and large language models. (B) Overview of AI applications across omics disciplines, highlighting its expanding role in multi‐omics integration, disease research, and the progression toward autonomous, intelligent systems. AI, artificial intelligence; DL, deep learning.

### AI‐driven multimodal data integration

The dynamic mapping of metabolite interactions under diverse physiological states helps elucidate disease mechanisms [148]. AI augments this process by introducing advanced computational capabilities that enhance every stage of the metabolomics workflow.

#### Frameworks for multi‐omics data integration and dynamic modeling

The integration of heterogeneous multi‐omics data demands AI architectures capable of reconciling technical and biological variability across modalities. Recent advances have led to computational frameworks to address the inherent heterogeneity of multi‐omics data [149]. Transformer‐based models, exemplified by scmFormer, leverage self‐attention mechanisms to harmonize single‐cell multi‐omics data, aiming to capture cross‐omics feature correlations and bridge technical gaps between modalities like the proteome and metabolome [150]. Similarly, the GLUE framework employs graph‐linked unified embeddings to align disparate omics feature spaces and construct cross‐omics cellular maps, showing capability in genome‐metabolome integration [99]. Beyond these, methods like the SIMO framework utilize probabilistic optimal transport to align spatial transcriptomics with other single‐cell modalities, supporting the reconstruction of cellular topologies and multi‐omics regulatory networks [151].

Static multi‐omics snapshots are often insufficient for unraveling disease progression dynamics. Bodein et al. [152] propose a hybrid network framework combining longitudinal multi‐omics data with node labeling and propagation analysis to identify context‐specific regulatory modules, such as kinase‐substrate networks governing cell differentiation. Parallel innovations include fuzzy smoothed embedding, which models nonlinear metabolite concentration changes during disease progression to allow for reinforcement learning‐driven metabolic flux prediction [153]. MaxFuse further enhances dynamic alignment through modality‐agnostic co‐embedding and probabilistic neighbor propagation, achieving 20–70% performance gains in feature linkage between weakly correlated modalities like spatial proteomics and transcriptomics. These methodologies underscore AI's capacity to disentangle biological variation from technical noise while capturing time‐resolved regulatory cascades, decoding time‐resolved metabolic reprogramming, and linking molecular events to phenotypic outcomes.

#### Knowledge‐augmented AI for interpretability

Bridging data‐driven AI with domain knowledge enhances biological plausibility and clinical translatability. The integration of explainable AI methods addresses model interpretability challenges, with systematic evaluations highlighting feature relevance and visual explanations as key tools for clinical translation [154]. Leng et al. [155] benchmark architectural choices in graph attention networks and variational autoencoders, demonstrating how attention mechanisms and latent space regularization optimize cross‐omics interaction modeling and modality alignment. The ENPKG framework integrates heterogeneous metabolomic data via graph neural networks to support drug discovery and chemical diversity characterization [156]. Network‐based approaches like PIUMet extend these capabilities by inferring molecular pathways from untargeted metabolomics data, identifying dysregulated metabolites even without experimental detection [157].

Practical implementation is streamlined through platforms such as MetaboAnalyst 5.0, which combines knowledge‐driven biological networks with AI‐powered dimensionality reduction, democratizes multi‐omics integration through enhanced lipidomics tools and user‐friendly workflows [158], while protocols like Ewald et al.'s [106] enable non‐experts to complete multi‐omics analyses in less than 2 h, achieving rapid, reproducible multi‐omics insights while addressing clinical translatability challenges. These innovations highlight the synergistic potential of embedding domain knowledge into AI‐driven workflows for enhanced biological interpretability.

### AI‐optimized metabolomics workflows

While these integrative AI frameworks enhance the translational potential of multi‐omics data, parallel advances in AI‐optimized workflows are critically improving the efficiency and accuracy of metabolomic data processing itself.

#### Automation for enhanced efficiency

AI is transforming metabolomics through the automation of traditionally labor‐intensive processes. DL‐based convolutional neural networks now address both monotonic and non‐monotonic retention time shifts in LC‐MS data, significantly improving chromatographic peak alignment accuracy across large sample cohorts [159]. To combat ion suppression effects, ML algorithms integrated with stable isotope internal standard libraries enable dynamic noise quantification and two‐stage quality control. This approach, combining random forest‐based noise identification with XGBoost‐driven signal correction, reportedly triples detection sensitivity for low‐abundance metabolites [160]. Furthermore, generative AI models like scGPT further revolutionize single‐cell multi‐omics integration, enabling tasks from cell typing to gene network inference through transfer learning on over 33 million cells [161].

Beyond these analytical enhancements, innovations in workflow automation driven by AI are further increasing throughput and strengthening data reliability. The SWATH‐MS workflow reduces false‐positive features by 40% through DL‐assisted peak prioritization [162], while the AutoBA framework leverages large language models to automate multi‐omics analysis via natural language interfaces, thereby drastically lowering technical barriers and enhancing reproducibility [163].

#### Precision‐driven metabolite annotation

AI‐driven frameworks offer the potential to enhance metabolite annotation by addressing key limitations in identification accuracy and coverage. For instance, the Knowledge‐Guided Multi‐Layer Network combines metabolic reaction networks, AI‐enhanced MS/MS similarity matching, and peak correlation networks, substantially improving annotation accuracy for known metabolites while also facilitating novel compound discovery [143]. ML methods such as METASPACE‐ML enhance imaging mass spectrometry by leveraging large‐scale training datasets to improve annotation precision at controlled false discovery rates [164]. Additionally, DL‐based tools, including DeepMSProfiler, enable end‐to‐end spectral feature extraction, mitigating batch effects and providing biologically interpretable metabolite‐protein correlations [165].

Beyond annotation, AI plays a crucial role in contextualizing metabolic data within multi‐omics landscapes and accelerating clinical translation. Advanced analytical pipelines integrate statistical and DL methods to combine cross‐sectional and longitudinal data, supporting robust biomarker discovery in complex diseases [166]. Cognitive computing approaches incorporate natural language processing to interpret metabolic features in biological contexts, thereby expediting mechanistic insight [167]. These approaches are complemented by open platforms such as OmicsPred, which employs genetic prediction models to facilitate large‐scale metabolic analysis without requiring costly experimental data [168].

These capabilities are further demonstrated in various disease‐specific applications, where ML identifies disrupted metabolic pathways and biomarkers in conditions including gout, cancer, metabolic syndrome, and chronic kidney disease [169, 170, 171, 172]. Predictive models also support personalized oncology and non‐invasive diagnostics, as shown in frameworks like DeepProg [173], and studies achieving high diagnostic sensitivity in gastric cancer detection [174]. Together, these advances underscore AI's essential role in translating multi‐omics data into clinically relevant and actionable outcomes.

### The AIVC: A key direction for metabolomics‐centric multi‐omics integration

The AIVC represents a transformative computational paradigm that integrates artificial intelligence with multi‐modal biological data to simulate the structure, function, and regulatory mechanisms of living cells at high resolution and fidelity [10]. Fundamentally, an AIVC functions as a digital twin of a real biological cell, a unified computational framework capable of simulating cellular and metabolic processes across spatiotemporal scales by dynamically integrating multi‐omics data with advanced cellular imaging [175]. The core architecture of an AIVC comprises three interconnected pillars: data integration, AI‐driven modeling, and predictive simulation. Within this architecture, metabolomics serves as a critical functional anchor, providing the closest readout of phenotype to constrain and validate model predictions [176].

The past year has witnessed remarkable advances across multiple frontiers of AIVC development. Large‐scale foundation models trained on tens of millions of single cells have achieved breakthrough performance in cell‐type annotation and gene‐function inference. The inaugural Virtual Cell Challenge marked a pivotal community effort, bringing together over 5000 participants to predict cellular responses to genetic perturbations. The competition demonstrated significant improvements in core capabilities such as perturbation discrimination and differentially expressed gene identification, while revealing that pure end‐to‐end learning has yet to fully solve the perturbation prediction problem [177]. Another landmark partnership has announced the generation of the largest perturbation dataset for virtual cell models to date, comprising over 120 million single‐cell data points across 225,000 drug‐patient interactions, representing one of the most ambitious biological data‐generation efforts in support of AI‐driven virtual biology [178].

Innovative approaches to AIVC construction have emerged across multiple scales of biological organization. At the tissue and organ level, frameworks like GigaTIME and HEX enable virtual staining and spatial proteomics from routine histopathology, facilitating population‐scale tumor immune microenvironment modeling and biomarker discovery that were previously infeasible due to data scarcity [179, 180]. At the multicellular level, a unified grammar framework translates human‐readable cell behavior descriptions into executable multi‐agent models, allowing in silico hypothesis testing and prediction of emergent cellular behaviors without requiring time‐series experimental data [181]. At the subcellular and single‐cell scale, generative models such as CellFluxV2 advance virtual cell representation, while patient‐specific Virtual Brain Twin frameworks integrate structural and functional data to enable non‐invasive diagnosis of neurological disorders [182, 183].

In biomedical research, these models simulate tumor metabolic reprogramming, guide targeted therapy design, and predict treatment efficacy in complex microenvironments. By simulating genetic perturbations, AIVCs predict functional roles in disease progression and, combined with CRISPR validation, facilitate drug target screening [184]. Foundational models such as Tahoe‐x1, trained on over 3 billion parameters, now enable the prediction of cancer cell responses to drugs and genetic perturbations at unprecedented scale and accuracy, demonstrating the potential of large‐scale AIVCs to reshape precision oncology and beyond [185]. However, the complexity of these models has intensified concerns regarding interpretability. Initiatives like the VCWorld model represent a shift toward “white‐box” simulators that integrate structured biological knowledge with large language models, generating interpretable, stepwise predictions alongside explicit mechanistic hypotheses [186]. This emphasis on interpretability aligns with broader calls to develop virtual cells that prioritize biological context and mechanistic understanding over sheer model scale [187].

Crucially, recent conceptual and methodological advances are addressing these challenges from multiple angles. Frameworks such as rbio1 now demonstrate the feasibility of “soft verification,” using virtual cell simulations to train and refine AI reasoning models without exclusive reliance on costly wet‐lab experiments [188]. This approach enables closed‐loop active learning systems that integrate in silico models with automated experimental platforms, forming intelligent agentic platforms that iteratively design, validate, and refine hypotheses through virtual‐physical feedback cycles [175]. Simultaneously, innovations in experimental technologies, such as ProPer‐seq for fixed‐cell perturbation profiling, are expanding the data ecosystem necessary for training and validating these next‐generation models [189]. By anchoring multi‐omics integration in the functional context of metabolism, AIVCs will ultimately enable predictive modeling of disease progression, personalized therapeutic design, and clinically actionable digital twins for precision medicine.

## 5 CHALLENGES AND PROSPECTS

The trajectory of any transformative technology in biomedical research often follows a predictable maturation process, commonly illustrated by the Gartner Hype Cycle, which delineates five phases: the innovation trigger, the peak of inflated expectations, the trough of disillusionment, the slope of enlightenment, and finally the plateau of productivity [190]. The integration of multi‐omics with artificial intelligence is currently navigating this curve. From these studies, we can derive principles for maximizing the output and validity of this promising research paradigm: it is crucial to navigate past initial hype through rigorous validation, reproducibility checks, and realistic goal‐setting to truly reach the plateau of sustained scientific productivity [190].

The application of artificial intelligence in multi‐omics exemplifies both the promise and the perils of this cycle. AI models require large, high‐quality, and well‐annotated datasets to achieve reliable predictions, yet multi‐omics data are often sparse, heterogeneous, and prone to batch effects [191]. Model interpretability remains another challenge. While AI can identify patterns, elucidating biologically meaningful mechanisms from these patterns is not always straightforward. Furthermore, many AI approaches operate as “black boxes,” complicating their adoption in clinical decision‐making where transparency is essential. These challenges represent the current “trough of disillusionment,” a phase where inflated expectations meet technical reality, demanding sustained effort to achieve meaningful breakthroughs.

Looking ahead, the path toward predictive, in silico biology, exemplified by the AIVC, is intrinsically linked to the adoption of a metabolomics‐centric framework. AIVC represents a pivotal direction for multi‐omics integration, serving as a unified computational framework capable of simulating cellular and metabolic processes across spatiotemporal scales. By dynamically integrating multi‐omics data, AIVC provides a powerful platform for deciphering complex biological mechanisms and enhancing clinical translation. We anticipate that continued development in this area will accelerate the convergence of virtual cell models with automated experimental systems, effectively forming intelligent agentic platforms that bridge in silico and in vitro environments. Such integration will enable iterative design‐validation cycles through closed‐loop wet‐dry workflows, creating a “virtual‐physical” feedback system that enhances reproducibility, refines predictive accuracy, and ensures biological relevance. These advances will establish a foundation for translational medicine, facilitating biomarker discovery, stratified therapy design, and ultimately paving the way toward clinically actionable digital twins.

## 6 CONCLUSION

In summary, this review has outlined the rationale for a metabolomics‐centered approach within multi‐omics research, underscoring its role in providing critical functional context for phenotypic interpretation. We have surveyed how artificial intelligence methodologies are advancing this field by enabling more sophisticated data integration and analysis. The logical progression of these converging developments points toward the construction of AIVC as a key objective. Achieving this goal will be crucial for building predictive models in systems biology and advancing translational precision medicine.

## AUTHOR CONTRIBUTIONS

**Ziyi Wang**: Writing—original draft; writing—review and editing. **Peng Chen**: Conceptualization; writing—review and editing; supervision. **Xiaoxu Wang**: Writing—review and editing; supervision. **Shiwen Deng**: Writing—review and editing; supervision. **Xiao Chang**: Writing—review and editing; validation. **Na Guo**: Writing—review and editing; validation. **Hongjun Yang**: Writing—review and editing; validation. All authors have read the final manuscript and approved it for publication.

## CONFLICT OF INTEREST STATEMENT

The authors declare no conflicts of interest.

## ETHICS STATEMENT

No animals or humans were involved in this study.

## Data Availability

No new data and scripts were used for this review. Supplementary materials (graphical abstract, slides, videos, Chinese translated version, and update materials) may be found in the online DOI or iMetaOmics http://www.imeta.science/imetaomics/.
